# Primary aldosteronism in pregnancy

**DOI:** 10.1007/s11154-022-09729-6

**Published:** 2022-05-10

**Authors:** Vittorio Forestiero, Elisa Sconfienza, Paolo Mulatero, Silvia Monticone

**Affiliations:** Division of Internal Medicine and Hypertension Unit, Department of Medical Sciences, University of Torino, Via Genova 3, 10126 Torino, Italy

**Keywords:** Primary aldosteronism, Pregnancy, Diagnosis, Management

## Abstract

Primary aldosteronism (PA) is the most common form of secondary hypertension. Although hypertensive disorders seem to affect around 5–10% of pregnancies worldwide, literature counts less than 80 cases of PA diagnosed during the peri-partum period. In this review we discuss about current knowledge on pathophysiology, natural history, diagnosis and treatment of PA in pregnancy. Because of the physiologic changes in the renin–angiotensin–aldosterone system (RAAS) and the contraindication to both confirmatory test and subtype differentiation, diagnosis of PA during pregnancy is challenging and relies mostly on detection of low/suppressed renin and high aldosterone levels. The course of pregnancy in patients with PA is highly variable, ranging from progesterone-induced amelioration of blood pressure (BP) control to severe and resistant hypertension with potential maternal and fetal complications. Mineralcorticoid receptor antagonists (MRA) are the recommended and most effective drugs for treatment of PA. As the anti-androgenic effect of spironolactone can potentially interfere with sexual development, their prescription is not recommended during pregnancy. On the other side, eplerenone, has proven to be safe and effective in 6 pregnant women and may be added to conventional first line drug regimen in presence of resistant hypertension or persistent hypokalemia. Ideally, patients with unilateral forms of PA should undergo adrenalectomy prior to conception, however, when PA is diagnosed during pregnancy and medical therapy fails to adequately control hypertension or its complications, adrenalectomy can be considered during the second trimester in case of unilateral adrenal mass at MRI-scan.

## Introduction

Primary aldosteronism (PA) is a heterogeneous group of disorders characterized by autonomous overproduction of aldosterone from the adrenal glands with subsequent increased sodium reabsorption and potassium excretion, low renin and arterial hypertension. PA can be distinguished into unilateral forms mainly caused by an aldosterone-producing-adenoma (APA), accounting for 30–40% of cases, and bilateral PA, which represents the remaining 60% of cases [1]. For many years PA prevalence has been underestimated since the presence of hypokalemia was considered essential for the diagnosis, while it is present at diagnosis in only a minority of patients [2]. Accumulating evidence overturned this assumption and PA is now recognized as the most frequent form of secondary hypertension, with an estimated prevalence of 6%-11% in hypertensive patients [1, 3], that progressively increases up to 20% in resistant hypertension [4].

Hypertensive disorders affect about 5–10% of pregnancies and represent a major cause of maternal, fetal and neonatal morbidity and mortality [5, 6]. Chronic or pre-existing hypertension, defined as systolic blood pressure (SBP) ≥ 140 mmHg and/or diastolic blood pressure (DBP) ≥ 90 mmHg before pregnancy or detected before the 20^th^ week of gestation, needs to be distinguished from new onset hypertensive complications of pregnancy, such as gestational hypertension and pre-eclampsia, which develop usually in the third trimester [7]. Based on large epidemiological studies, chronic hypertension is estimated to affect 1.3–1.8% of pregnancies, a prevalence that has significantly increased in the last 4 decades by almost 6% per year; in addition, the rate of chronic hypertension increases steadily with maternal age and is twofold higher in black women [8, 9]. The large majority of the described cases of chronic hypertension in pregnancy were due to essential hypertension, with a prevalence of 1.52%, but secondary forms due to endocrine and renal disorders, although rare (prevalence of 0.24%), were associated with a greater risk of adverse maternal and fetal outcomes [8].

Few data are currently available on prevalence, diagnosis and management of PA during pregnancy, mostly derived from single case reports or studies conducted on a limited number of patients. A recent English cohort study considering almost 3 million pregnancies over a four-year period identified only 3 cases of PA diagnosed before or during pregnancy [10]. However, assuming a 5–6% PA prevalence in the general hypertensive population, we could speculate that 0.6–0.8% of all pregnant women would suffer from PA [11]. Nevertheless, since the first report described by Crane et al. in 1964 [12], excluding familial forms, less than 80 cases of pregnant women with PA have been reported [11, 13, 14], suggesting that PA is significantly underdiagnosed prior to and during pregnancy. This assumption is even more realistic considering that a larger number of Cushing’s syndrome and pheochromocytoma cases have been described in pregnant patients, despite being fewer common causes of secondary hypertension in the general population [15, 16]. Despite guidelines recommendations [2, 17, 18], the screening rate for PA in the hypertensive population is very low [19, 20] and no specific formal recommendations for pregnant patients with PA have currently been published.

The aim of this review is to summarize current knowledge on physiopathology, natural history, diagnosis and management of PA during pregnancy.

## Pathophysiology

**Fig. 1:**
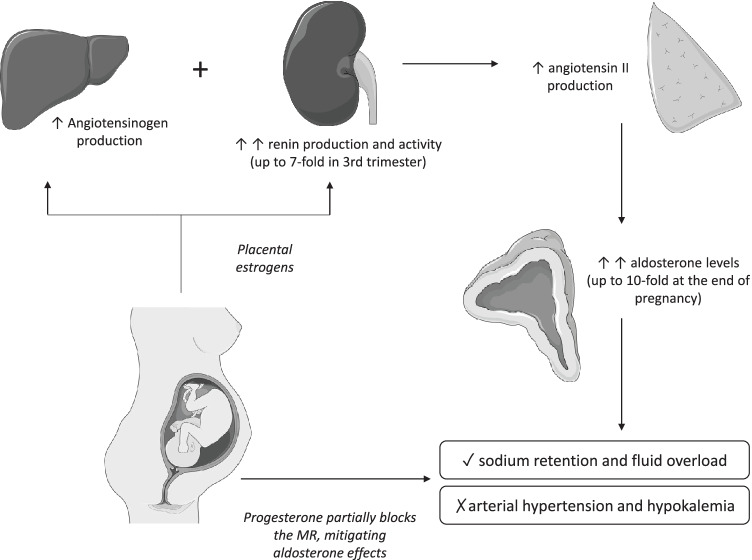
Renin–angiotensin–aldosterone system (RAAS) changes in physiologic pregnancy. MRs: mineralcorticoid receptors. The figure was generated using medical images from https://smart.servier.com/

Physiologic pregnancy is characterized by dramatic changes in a number of endocrine systems and particularly by an overall upregulation of the renin–angiotensin–aldosterone system (RAAS), which plays an important role in salt balance and subsequent well-being of mother and fetus (Fig. 1) [21, 22].

Estrogens produced by the growing placenta stimulate hepatic synthesis of angiotensinogen and corticosteroid-binding globulin (CBG), the primary serum carrier protein of cortisol [23].

The increased angiotensinogen production, along with renal estrogen stimulation and the presence of extra-renal synthesis by ovaries and maternal decidua [24] leads to an important increase in both plasma renin concentration and activity, the latter being estimated to be fourfold at the 10th week of gestational age (GA) and almost sevenfold at term [25].

Increased renin activity promotes increased angiotensin II levels, which stimulates aldosterone production in the zona glomerulosa. Aldosterone levels reach concentrations tenfold higher than baseline by the end of pregnancy, resulting in sodium retention and fluid overload, essential to obtain adequate placental perfusion [26]*.* Nevertheless, pregnant women do not normally exhibit symptoms of hyperaldosteronism such as hypertension or hypokalemia, mainly because progesterone, whose levels progressively increase during pregnancy, acts as a competitor of aldosterone in the distal convoluted tubule, blocking the mineralcorticoid receptors and thus attenuating aldosterone effects [27, 28]. Another possible explanation is the development of resistance in the maternal vessels to the vasopressor effect of angiotensin II. This is thought to be due to the presence of increased progesterone and prostacyclins, which can decrease angiotensin receptor type 1 (AT1) sensitivity [29].

In addition to aldosterone, the levels of deoxycorticosterone, another mineralcorticoid mostly deriving from extra-adrenal 21-hydroxylation of circulating progesterone, markedly increase during pregnancy. In light of these hormonal changes, how the large majority of women remain normotensive during pregnancy remains unclear [30].

The aberrant expression of luteinizing hormone (LH) and/or gonadotropin releasing hormone (GnRH) receptors in the adrenal zona glomerulosa may lead to aldosterone overproduction and such effect may be more pronounced during pregnancy due to the high levels of human chorionic gonadotropin [31, 32]. The first *in vivo* demonstration of aldosterone responsiveness to gonadotropin stimulation in a pregnant woman with PA was described by Albiger et al. who showed a significant increase of aldosterone levels (114%) after a GnRH injection. After adrenalectomy the APA displayed overexpression of GnRH/LH receptors [33]. In the same study partial or significant response to the GnRH test was found in other 12 non-pregnant patients with APA, while healthy control subjects showed no increase in aldosterone levels. More recently, somatic-activating mutations of CTNNB1, involved in the ß-catenin/WNT signaling pathway, were identified in APA from two pregnant women and one postmenopausal woman, and suggested to be responsible for GnRH/LH receptors overexpression [34]. This hypothesis was partially rejected in a larger cohort of patients in which, although aberrant regulation of aldosterone by GnRH or LH stimulation was present in at least 50% of cases, no association with CTNNB1 mutations was identified [35]*.*

Pre-eclampsia (PE) is a pregnancy-related complication defined as the new onset of hypertension after the 20th week of gestation associated with proteinuria ≥ 300 mg/24 h and/or maternal kidney failure, liver dysfunction, neurologic symptoms, hemolysis or thrombocytopenia [6]. Although its etiology is still unknown, both up-regulation of RAAS and systemic endothelial dysfunction due to the over-production of pro-inflammatory factors from the placenta have been indicated as potential pathogenic factors [36].

Studies on women with pre-eclampsia showed a high prevalence of angiotensin II type 1 receptor autoantibody (AT1-AA) [37, 38], which are responsible for an increased sensitivity to angiotensin II and a higher risk of hypertension and proteinuria development as demonstrated in animal models [36, 39], beside other vascular effects such as vasoconstriction of small arteries [40], production of reactive oxygen species (ROS) and promotion of antiangiogenic factors secretion [41].

More recently, high levels of AT1-AA were identified in patients with PA and were proposed as a possible mechanism enhancing excessive aldosterone production [42], despite contrasting results in terms of plasmatic levels between APA and bilateral PA among the studies [43–47].

In conclusion, although several hormonal changes and potential mechanisms have been described, further studies are needed to clarify the potential relationship between gonadotropin stimulation and PA development during pregnancy as well as between PE, PA and AT1-AA.

## Natural history

The course of PA during pregnancy is highly variable, ranging from worsening of BP control and hypokalemia, which may lead to severe fetal and maternal complications [21], to improvement both of BP levels and biochemical abnormalities [28, 48–50]. Although not completely understood, the reasons for this heterogeneous course may reside in PA severity. When aldosterone exceeds its normal pregnancy-related concentrations, the mitigating effect of progesterone is insufficient to prevent the development of hypertension and hypokalemia [50]. On the other hand, in mild forms of PA, the higher progesterone levels occurring in pregnancy seem to contrast aldosterone effects, leading to improvement in BP control.

Supporting evidence derives from case reports. In 2011 Ronconi et al. described a case of a hypertensive woman with PA reporting normal BP values throughout the entire course of pregnancy but experiencing a sudden worsening of BP status soon after delivery, due to the drastic decrease of progesterone levels associated with persistently high aldosterone [28]. Similarly, a new onset of hypertension in the post-partum period led to PA diagnosis in several patients, who showed high BP values and hypokalemia with suppressed renin and high aldosterone levels, mostly due to unilateral forms of PA [51–55]. In fact, the progressive decrease in progesterone levels and the increased levels of prolactin during pregnancy and the peri-partum period may unveil an unknown APA in previously normotensive women [55, 56]. In particular, aldosterone producing adenoma were found to express higher levels of prolactin receptors when compared to normal adrenal glands, suggesting a role of prolactin in the stimulation of aldosterone secretion [57].

Systematic reviews reported a higher incidence of pregnancy-related complications in pregnant women with PA when compared with the general obstetric population [21, 58]. In particular, pre-eclampsia (PE) affects almost one third of pregnant patients with PA, but the incidence reported in patients with PA, despite being 3 times higher than that observed in the general population, does not differ significantly from pregnant patients with essential hypertension [6]. Furthermore, PE may lead to other pregnancy-related complications such as fetal growth abnormalities, pre-term birth and delivery complications. In the available case reports, almost half of pregnancies developed pre-term birth, with a mean gestational age at delivery of 33 weeks, while caesarean section for either uncontrolled hypertension, severe intra-uterine growth restriction (IUGR) or maternal complications was performed in half of the cases [21]. Such alarming data were confirmed in a very recent European retrospective study conducted on 19 pregnancies in patients diagnosed with PA after delivery [13]: PE was the most frequent complication and occurred in 26% of the cases, pre-term delivery occurred in 5 cases, and more than half of the patients underwent cesarean section, mainly due to fetal distress secondary to either PE or uncontrolled maternal BP values. However, considering that mild forms of PA may be unrecognized during pregnancy, the statistics above are probably affected by the most severe forms and therefore may not reflect the real scenario. Furthermore, hypokalemia itself can be responsible for fearsome maternal complications like cardiac arrhythmias and paralysis. On the other hand, an extensive review of the literature, including nearly 100 pregnant patients with Bartter or Gitelman syndrome, excluded direct negative consequences of maternal chronic hypokalemia on fetal development, thanks to the stable potassium levels of the fetus ensured by the activity of the maternal–fetal transport of potassium across the placenta [59].

Women with familiar hyperaldosteronism type 1 (FH-1) seem to show a more benign course of pregnancy, with a rate of maternal complications, such as pre-eclampsia, and fetal outcomes, similar to that of the general population [60, 61]. FH-1, also called glucocorticoid-remediable aldosteronism, is a monogenic form of PA due to the chimeric *CYP11B1/CYP11B2* gene, encoding for a chimeric enzyme that produces aldosterone under ACTH control and requires treatment with low-dose dexamethasone to control blood pressure. A possible explanation for the better outcome in this specific PA subtype is the presence of a direct inhibitory effect of progesterone on the chimeric CYP11B1/CYP11B2 enzyme, as demonstrated *in vitro* [62].

Nevertheless, most of this information derives from case reports and needs to be confirmed in larger cohorts and/or controlled longitudinal studies comparing patients with PA *versus* normotensive and/or women affected by chronic essential hypertension in terms of pregnancy-related complications and course.

## Diagnosis

According with Endocrine Society guidelines and ESH consensus, the diagnosis of PA requires an initial screening test consisting in the determination of the aldosterone-renin ratio (ARR), followed by confirmatory test and subtype differentiation [2, 17, 63]. The lack of pregnancy-specific reference ranges and the significant changes in the renin-angiotensin system occurring during pregnancy make the diagnosis of PA in pregnant women particularly challenging. Both renin and aldosterone serum levels are increased during pregnancy, but the earlier and proportionately greater increase in renin activity may lower the ARR, resulting in a greater number of false negatives [17]. Nevertheless, systematic reviews of the literature show that pregnant women with PA usually maintain lower plasma renin activity (PRA) levels compared to either normotensive individuals or pregnant subjects with essential hypertension, being suppressed in approximately 60% of the described cases [58]. PRA less than 4 ng/ml/h together with high plasmatic aldosterone concentration (PAC) and hypokalemia have been proposed as a high suspicion criterion for PA in pregnant women with hypertension [64]. More recently, an Australian retrospective study enrolling 9 pregnant subjects diagnosed with PA and 33 pregnant women with chronic hypertension showed how direct renin concentrations (DRC) were significantly lower and ARR was significantly higher in the PA group, while no differences were found in the aldosterone levels: the combination of an ARR > 40 pmol/L/mU/L and DRC < 20 mU/L was therefore proposed as suggestive for the diagnosis of PA during pregnancy [14].

When screening test is performed, it is crucial to avoid assumption of interfering medications. In particular, labetalol and α-methyldopa, blocking sympathetic-induced renin secretion, can lead to false positive tests and should be withdrawn [17]. Despite a potential risk of false negative results, nifedipine, due to its safety profile, represents a valid option for ARR testing during pregnancy. If blood pressure is not controlled by nifedipine alone, hydralazine can be added [17].

**Fig. 2:**
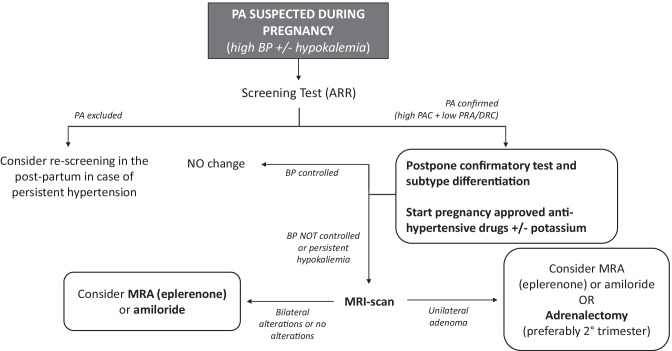
Diagnosis and management of PA during pregnancy. PA: primary aldosteronism; BP: blood pressure; ARR: aldosterone/renin ratio; PAC: plasma aldosterone concentrations; PRA: plasma renin activity; DRC: direct renin concentration; MRA: mineralcorticoid receptor antagonists; MRI: magnetic resonance imaging

Suspicion of PA during pregnancy should arise in presence of moderate to severe hypertension, especially if occurring before the 20th week of gestation, with associated hypokalemia (Fig. 2) [65]. In fact, although hypokalemia is not a sensitive tool for PA screening in the general population [17], serum potassium levels have found to be low in the majority of described cases of PA during pregnancy [13, 58]. However, the anti-mineralcorticoid effect of progesterone and the physiologic pregnancy-related metabolic acidosis may mask hypokalemia in milder phenotypes of PA [66].

In patients with suspicion of PA but negative ARR during pregnancy, screening test should be repeated in the post-partum period (Fig. 2) [58]. Since both PRA and aldosterone decrease 6 weeks after delivery [67] and the estrogens levels also sharply decrease after placental expulsion, the first months of the post-partum are appropriate for pursuing PA diagnosis. Finally, the high prolactin levels detected during the breast-feeding are not known to specifically interfere with ARR determination and may even promote PA detection, as previously described.

To avoid the risk of excessive fluid overload or teratogenic/toxic effects, confirmatory tests with saline infusion or captopril challenge test are not routinely recommended during pregnancy.

Regarding the subtype diagnosis, imaging test with MRI or ultrasonography can be performed during pregnancy to detect adrenal masses, but adrenal CT-scan and adrenal venous sampling (AVS) should be avoided, due to radiation exposure. Therefore, in case of satisfactory BP control with pharmacological therapy, it is highly recommended to postpone the diagnostic work-up after the delivery (Fig. 2) [11]. In patients with clinical suspect of PA and severe or resistant hypertension, imaging of the adrenal glands with MRI should be performed. In case of severe and uncontrolled hypertension, pregnant women with a solitary unilateral adrenal lesion suspected for cortical adenoma and biochemical values strongly suggestive for PA (marked aldosterone excess with serum values > 30 ng/dL, undetectable renin and spontaneous hypokalemia), because of their young age, can be directly referred for adrenalectomy without performing neither confirmatory test nor AVS [17, 63]. In contrast, if screening tests are still not conclusive or a bilateral disease is suspected at imaging test, the only available option is to revise medical treatment (Fig. 2). When adrenalectomy is considered, it is mandatory to exclude concomitant cortisol co-secretion from the adrenal adenoma: as the hypothalamic–pituitary–adrenal axis is overall upregulated and suppression of cortisol levels by exogenous glucocorticoids is lower during pregnancy, the recommended approach is the combined use of urinary free cortisol and late-night salivary cortisol, with specific reference values for pregnancy [65].

## Treatment

Currently no formal recommendations for the therapeutic management of pregnant women with PA have been proposed due to the lack of randomized-control trials or large-scale cohort studies. Regardless of treatment strategy, the key point of management is to achieve blood pressure (BP) control, in order to prevent hypertension-associated maternal and fetal morbidity and mortality, and to correct hypokalemia with oral and, if needed, intravenous potassium supplementation [68].

The European Society of Cardiology (ESC) Guidelines recommend starting pharmacological treatment during pregnancy in presence of SBP ≥ 150 mmHg and/or of DBP ≥ 95 mmHg. In women with gestational hypertension, pre-existing hypertension with the superimposition of gestational hypertension or hypertension and subclinical organ damage, drug treatment should be initiated in presence of SBP ≥ 140 mmHg and/or DBP ≥ 90 mmHg [7].

BP target for hypertension control during pregnancy is still not know and ESC guideline does not provide any recommendation on this regard. Data from a multicenter trial enrolling almost one thousand pregnant women with chronic or gestational hypertension showed no differences in terms of incidence of pregnancy-related complications between patients more or less intensively treated (group 1: DBP target < 85 mmHg *vs* group 2: DBP target < 100 mmHg), despite a higher risk of severe hypertension status in the second group [69]. In view of these findings, a DBP ranging from 80 to 85 mmHg may represent a valid goal [70], and a larger, currently ongoing, multicentric randomized controlled trial may provide confirmation in the next future [71].

α-methyldopa, beta blockers (mostly labetalol) and calcium antagonists (mostly nifedipine) are the first-line recommended antihypertensive drugs in pregnancy because of their proven safety and efficacy [7], while ACE-inhibitors and angiotensin-receptor blockers (ARBs) are strictly forbidden for their teratogenicity [7]. Despite mineralcorticoid receptor antagonists (MRAs) represent the first line choice for PA treatment, no strong data on their safety during pregnancy are currently available.

Spironolactone was the first MRA to be introduced in 1960 and was widely prescribed in pregnant women for the treatment of hypertension, preeclampsia, liver disease and myasthenia gravis until 1980, when Hecker et al. raised doubts on spironolactone-induced feminization in male rat fetuses after treatment of the mother with high dose of spironolactone in the first days after conception [72]. Considering that genital differentiation takes place in the first trimester, spironolactone treatment in early pregnancy can potentially cause feminization of a male fetus, because of its well-known anti-androgenic effect and its ability to cross the placenta*.* A recent extensive analysis of the published data reported that spironolactone-induced feminization was mentioned in 6 of 9 studies on animals and almost all the anti-androgenic effects were observed in animals exposed to human equivalent doses of more than 200 mg per day, which is significantly higher than the dosage usually prescribed to control PA (around 50–100 mg per day) [73]*.* To our knowledge, among humans only one case of spironolactone-induced sexual-ambiguity in a male newborn from a mother under spironolactone-treatment from the beginning till the 5th week of gestation has been described [74]. By contrast, there are several cases in which spironolactone was prescribed to mothers with PA, Bartter and Gitelman syndromes with no adverse effects on the newborns, though the medication was used in the first trimester in only one case [11, 75, 76]*.* In a very recent case report of a pregnant woman with resistant hypertension secondary to both Cushing syndrome and PA, spironolactone was prescribed during the second trimester after determination of the female sex of the fetus [77].

In conclusion spironolactone is routinely contraindicated during pregnancy due to the lack of evidence on its safety in controlled trials and its prescription should be therefore discussed case by case according to pros (benefit in terms of BP control and hypokalemia correction) and cons (risk for anti-androgenic effects), despite an apparent low risk for the fetus when it is prescribed at a low dose and after the first trimester of pregnancy.

Eplerenone is a selective MRA showing lower affinity for androgen receptors when compared with spironolactone [78]. Pregnant rats and rabbits exposed to doses of eplerenone 30 times higher than those normally used in humans experienced no teratogenic effects [79]. Eplerenone was prescribed in 6 pregnant women with either PA or Gitelman syndrome, diastolic hearth failure and resistant hypertension with severe obstructive-sleep-apnea-syndrome (OSAS), without evidence of sexual ambiguity [80–85]. These data allow to consider eplerenone a relatively safe drug which may be considered as a valid option for resistant hypertension in pregnant women with PA, although the lack of evidence deriving from large studies on humans still reserves eplerenone as a class B medication in pregnancy.

Blockers of the sodium epithelial channel (ENaC), like amiloride and triamterene, are currently not commonly used in pregnancy because of the lack of appropriate safety studies and the potential risk of hypovolemia. Animal studies using high dosages of amiloride revealed no evidence of side effects in the fetus [86]. Furthermore, amiloride has been prescribed in 8 pregnant patients with PA [10, 34, 87–91] without apparently side effects. Therefore, as eplerenone, amiloride could be considered for treatment of resistant hypertension in patients affected by PA during pregnancy.

**Fig. 3:**
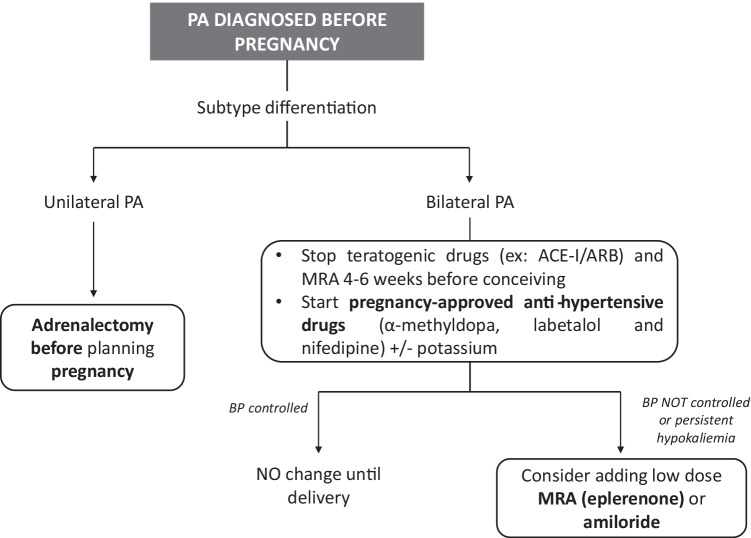
Management of a patient with PA and desire of pregnancy. PA: primary aldosteronism; ACEI: ACE-inhibitors; ARB: angiotensin-receptor blockers; MRA: mineralcorticoid receptor antagonists; BP: blood pressure

According with PA guidelines, young patients with spontaneous hypokalemia, marked aldosterone excess and unilateral adrenal lesions suggestive for cortical adenoma may avoid AVS and proceed directly to unilateral adrenalectomy [17, 63]. In case of pregnant women with uncontrolled BP and persistent hypokalemia, the more reasonable option is to perform surgery in the second trimester of pregnancy because of the lowest risk for fetal and maternal complications (Figs. 2 and 3) [92]. Eleven cases of patients with PA who underwent adrenalectomy during pregnancy are described in literature: the main indication was the presence of uncontrolled BP values, despite optimized antihypertensive therapy, and persistent hypokalemia in presence of a unilateral adrenal mass [83, 88, 92–100]. After adrenalectomy, almost all reported cases showed partial clinical response with improvement of BP control and reduction of number of anti-hypertensive drugs, while complete response was achieved in one case [92]. Regarding biochemical response, renin and aldosterone values were reported after adrenalectomy in one case and showed normalization of the aldosterone-renin-ratio [98]. In seven of the reported cases serum potassium levels normalized after adrenalectomy, in one case mild hypokalemia persisted in the immediate postoperative, while no cases of hyperkaliemia secondary to contralateral adrenal’s suppression are reported. Zona glomerulosa insufficiency has been described as a cause of transient or persistent hyperkaliemia after adrenalectomy in patients with APA, related to chronic renin suppression [101, 102]. As for non-pregnant patients, it is mandatory to routinely measure electrolytes within the initial period after adrenalectomy [17]. On the other hand, in the described cases of adrenalectomy during pregnancy, a high rate of adverse outcomes was observed, with five women experiencing either intra-uterine fetus growth retardation (IUGR) or pre-eclampsia, with subsequent pre-term induced delivery. In a single case intrauterine fetal death was diagnosed at 26th week of gestation, as a consequence of severe IUGR: on histological examination a marked thickening of the placental artery was found, suggesting irreversible changes in the placental vessels due to the high BP values leading to insufficient blood flow to the fetus [97].

## Conclusion

In conclusion, the following recommendations for pregnant women with PA can be proposed (Figs. 2 and 3): (i) in women with a confirmed diagnosis of PA, conception should be planned in order to schedule a more strict and appropriate follow-up; (ii) women with unilateral forms of PA and at childbearing age with a desire of motherhood should be addressed to adrenalectomy, before planning pregnancy; (iii) in case of idiopathic hyperaldosteronism, treatment with MRAs and potentially teratogenic anti-hypertensive drugs should be stopped 4–6 weeks before conception and treatment with first-line medications (such as α-methyldopa, labetalol and nifedipine) should be started; (iv) in presence of idiopathic PA with severe hypertension and hypokalemia, a switch from spironolactone to eplerenone at the lowest effective dose could be considered; (v) when PA is diagnosed during pregnancy, proper anti-hypertensive therapy with first-line drugs should be started, postponing confirmation test and subtype diagnosis after delivery; (vi) in case of poor BP control and/or refractory hypokalemia, eplerenone or amiloride might be considered, while spironolactone should be avoided, especially in the first trimester, because of its anti-androgenic side effects; (vii) finally, in patients with APA and resistant hypertension despite optimized anti-hypertensive therapy, especially in presence of initial signs of pregnancy-related complications, laparoscopic adrenalectomy could be considered in the second trimester of pregnancy in order to avoid severe maternal and/or fetal complications.
